# Sequencing data and MLPA analysis data in support of the effectiveness and reliability of an asymmetric PCR-Based approach in preparing long MLPA probes

**DOI:** 10.1016/j.dib.2015.05.008

**Published:** 2015-05-27

**Authors:** Xingyuan Ling, Hai Long, Guang Pan, Zhinan Chen

**Affiliations:** a Animal and Plant Inspection and Quarantine Technical Center, Shenzhen Entry-Exit Inspection and Quarantine Bureau, 1011 Fuqiang Road, Futian District, Shenzhen City 518045, Guangdong Province, China; b Institute of Animal and Plant Inspection and Quarantine, Shenzhen Academy of Inspection and Quarantine, 2049 Heping Road, Luohu District, Shenzhen City 518001, Guangdong Province, China

**Keywords:** MLPA probe, DNA sequencing, MLPA analysis, ABI PRISM 3100 Genetic analyzer

## Abstract

ABI PRISM 3100 Genetic Analyzer, a multi-color fluorescence-based DNA analysis system with 16 capillaries operating in parallel, was ideal tool both for DNA sequencing and DNA fragment analysis [1,2]. To demonstrate the effectiveness and reliability of an asymmetric PCR-Based approach (X.Y. Ling, G.M. Zhang, G. Pan, H. Long, Y.H. Cheng, C.Y. Xiang, L. Kang, F. Chen, Z.N. Chen, Preparing long probes by an asymmetric PCR-based approach for multiplex ligation-dependent probe amplification (MLPA), Anal. Biochem. (2015), http://dx.doi.org/10.1016/j.ab.2015.03.031, in press) in preparing the long MLPA probes that were generated with a M13-based method before [4], some prepared long MLPA probes were sequenced and then tested in MLPA analysis. Sequencing data shows that the long MLPA probes were identical to the designed ones, indicating the long probes can be easily prepared with the new method, and the MPLA analysis data shows that the results of MPLA analysis with these long probes were as same accurate and specific as with ones prepared with other methods. The sequencing data was not presented in the research article (X.Y. Ling, G.M. Zhang, G. Pan, H. Long, Y.H. Cheng, C.Y. Xiang, L. Kang, F. Chen, Z.N. Chen, Preparing long probes by an asymmetric PCR-based approach for multiplex ligation-dependent probe amplification (MLPA), Anal. Biochem. (2015), 10.1016/j.ab.2015.03.031, in press), but the MLPA analysis data was converted into figure 4 and figure 5 of the research article.

Specifications Table

**Table:** 

Subject area	Biology
More specific subject area	Preparation method of long single-strand DNA probe
Type of data	figure
How data was acquired	3100 Genetic analyzer
Data format	Raw and processed
Experimental factors	DNA sequencing was done by a company (Shenzhen Huada Gene Research Institute). The probe sequences were analyzed from only one direction by using the common reverse primer MLPA-R [3]. For fragment analysis, a 10 μl of sample mixture containing 1 μl of the MLPA product, 8.7 μl of HiDi formamide and 0.3 μl of Genescan 500 LIZ size standard was prepared. The mixture was denatured for 5 min at 95° and then immediately cooled on ice. ABI PRISM 3100 Genetic analyzer was set up by using 36-cm capillary and POP-4-polyner, and 3100 Data Collection Software under GeneScan mode was selected.
Experimental features	The sequencing raw data was viewed and a bitmap was copied with software BioEdit Sequence Alignment Editor. All MLPA analysis data (raw fragment analysis data) was collected with Data Collection Software, but viewed and analyzed with GeneScan 3.7 software, and finally the figures of these analyses were screen captured.
Data source location	Shenzhen City, Guangdong Province, China.
Data accessibility	These data are with this article.

## 1 Value of the data

• These sequencing data attached were the direct evidences confirming that the prepared long probes were identical to the designed ones, thereby proving that the described method [3] is high effective and reliable in preparing the long single-strand MLPA probes.
• The MLPA analysis data, which showed that the prepared long probes were as same effective and reliable in MLPA analysis as the ones prepared with other methods, was also valuable to researchers who are interested in developing the multiplex MLPA analysis as a detection module for GM maize detection.
• The data is not only helpful for the researchers to understand and evaluate the value and advantage of the described method [3] in preparing the long single-strand DNA probe, but also be valuable to researchers to consult the strategy of this described method to prepare the similar long single-strand DNA probes for other purpose.

## 2 Data, experimental design, materials and methods

### 2.1 Sequencing analysis

Following the procedure of the report [3], the long 3′ hemi-probe for GM maize event MON810, NK603, MON863, MON89034, MON88017, MIR604, GA21, BT11, 59122, 3272, CBH351 and LY038 were prepared. To check whether the right stuffer sequences of pUC18 were introduced into the long 3′ hemi probes in right site, seven long 3′ hemi-probes respectively for GM maize event MON863, MON89034, MON88017, 59122, 3272, CBH351 and LY038, were subjected to sequencing. Because the sequencing work was highly commercial, the seven probe sequences were determined by Shenzhen Huada Gene Research Institute by using primer 5′–GCGCCAGCAAGATCCAATCTAGA-3′ (MLPA-R). The raw sequencing data was viewed and analyzed with software BioEdit Sequence Alignment Editor. For each prepared probe, a bitmap of about 100 bp was copied to show that the selected unrelated sequence of pUC 18 was introduced in the right site of the long MLPA probe. The seven bitmaps were not presented in the published research article [3], but could be accessed from the supplementary data (supplementary Figs. 1–7). The detail sequences of the 3′long hemi probe for MON863, MON89034, MON88017, 59122 were listed in table 2 of the research article [3].

### 2.2 Fragment analysis of MLPA product

Totally, three simplex MLPA analyses and three multiplex MLPA analyses were carried out according to the procedure of the research article [3]. MLPA analyses products are DNA fragments with difference length, which can be easily separated and be determined by Genetic analyzer. To analyze the MLPA product with 3100 Genetic analyzer, a 10 μl of mixture containing 1 μl of the MLPA product, 8.7 μl of HiDi formamide and 0.3 μl of Genescan 500 LIZ size standard was prepared, and 3100 Genetic analyzer was set up with 36-cm capillary and POP-4-polymer. The sample mixture was denatured for 5 min at 95° and then immediately cooled on ice before it was loaded to the instrument that was driven by Data Collection Software under GeneScan model. All raw electropherogram data were opened and analyzed with GeneScan 3.7 software and the corresponding figures were screen captured by pressing the “printer/schem” button and stored in a word pad file by pushing “control/v” button. The six raw figures of the six MLPA analyses could be accessed from the supplementary data (supplementary Figs. 8–13), they were converted into figure 4 and figure 5 of the research article [3] to show that the size of MLPA analyses products are fully identical to the expected ones listed in table 2 of the research article, and thereby to prove that the probes prepared with the asymmetric PCR-Based approach are as same reliable in MLPA analysis as the ones prepared with other methods.

## Footnotes

Appendix A Supplementary data associated with this article can be found in the online version at doi:10.1016/j.dib.2015.05.008.
