# Supplementary material for: Sequencing data and MLPA analysis data in support of the effectiveness and reliability of an asymmetric PCR-Based approach in preparing long MLPA probes
Source: Data Brief. 2015 May 27;4:203–6. doi: 10.1016/j.dib.2015.05.008 (PMC4510450; doi:10.1016/j.dib.2015.05.008)
Supplement: Supplementary file 1 — Supplementary data [file mmc1.doc]

**Part one:** Bitmaps of partial sequencing raw data of seven preparted long 3’ hemi-probes showing the sequences of these prepared probes were identical to the designed ones and the stuffer sequences of pUC18 were introduced into the long probes at the right site listed in table 2 of the research article [3]


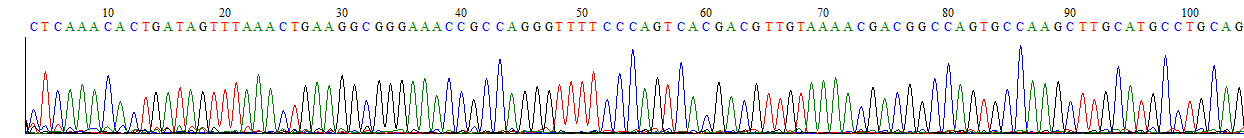


**Figure 1:** Bitmap of partial sequencing data of long 3’ hemi-probe 59122-2


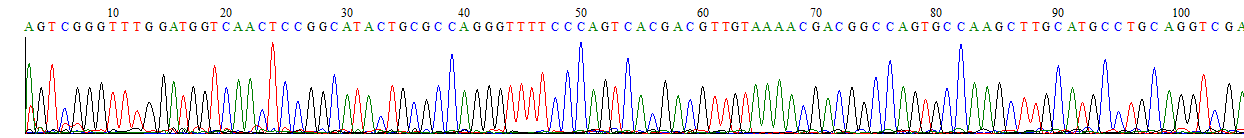
 **Figure 2:** Bitmap of partial sequencing raw data of long 3’ hemi-probe MON88017-2


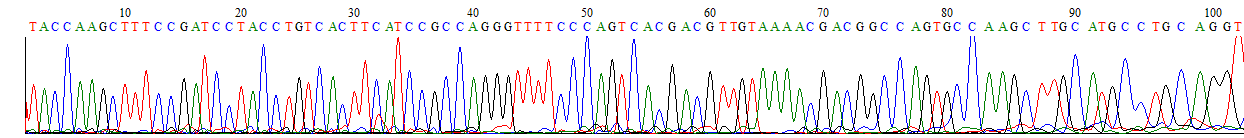
 **Figure 3:** Bitmap of partial sequencing raw data of long 3’ hemi-probe MON863-2)


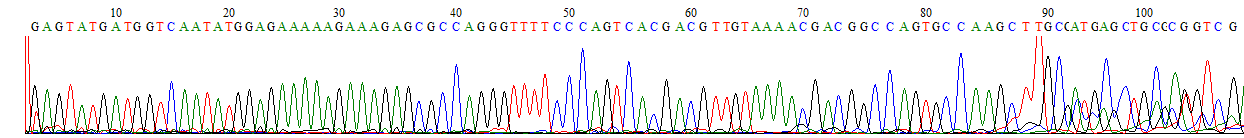


**Figure 4:** Bitmap of partial sequencing raw data of long 3’ hemi-probe MON89034-2


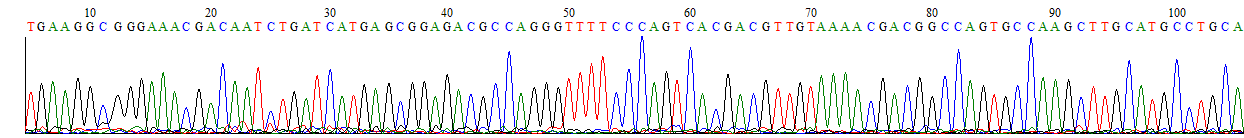
 **Figure 5:** Bitmap of partial sequencing raw data of long 3’ hemi-probe 3272-2


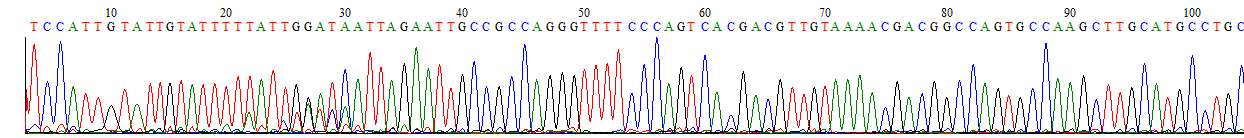


**Figure 6:** Bitmap of partial sequencing raw data of long 3’ hemi-probe CBH351-2


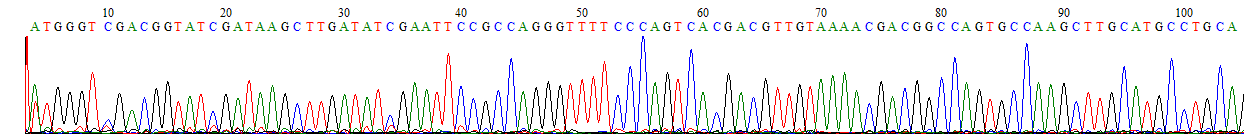


**Figure 7:** Bitmap of partial sequencing raw data of long 3’ hemi-probe LY038-2

**Part two:** Results of three simplex MLPA analyses and three multiplex MLPA analyses showing the size of MLPA analyses products are fully identical to the expected ones listed in table 2 of the research article [3]


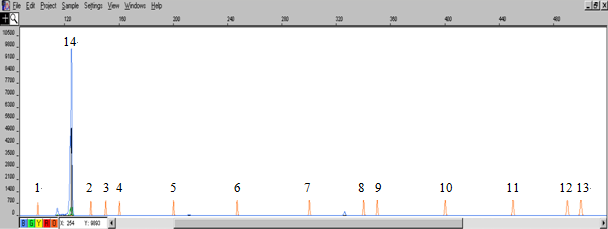


**Figure 8:** Results of simplex MLPA analysis with the probe set for GM maize event TC1507. The product was 109bp. Peak 1-13: GeneScan^TM^ 500 LIZ Size Standard, 100bp, 139bp, 150bp, 160bp, 200bp, 250bp, 300bp, 340bp, 350bp, 400bp, 450bp, 490bp and 500bp respectively; peak 14 in Fig. 4(a), (b) and (c): 109bp, 211bp and 271bp respectively.


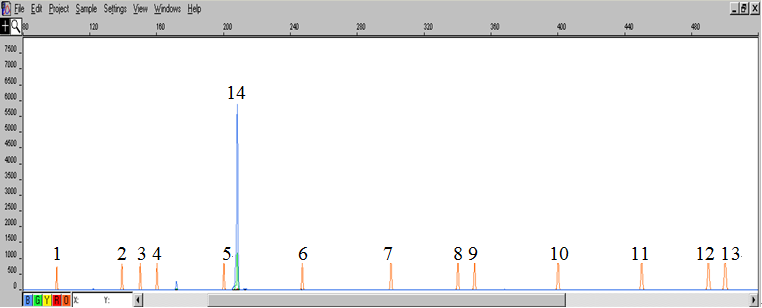


**Figure 9:** Results of simplex MLPA analysis with the probe set for GM maize event MON860. The product was 211bp long. Peak 1-13: GeneScan^TM^ 500 LIZ Size Standard, 100bp, 139bp, 150bp, 160bp, 200bp, 250bp, 300bp, 340bp, 350bp, 400bp, 450bp, 490bp and 500bp respectively; peak 14 in Fig. 4(a), (b) and (c): 109bp, 211bp and 271bp respectively.


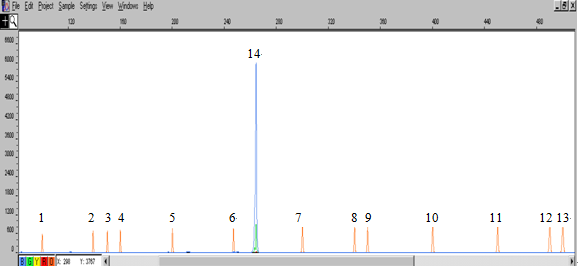


**Figure 10:** Results of simplex MLPA analysis with the probe set for GM maize event MIR604. The product was 271bp long. Peak 1-13: GeneScan^TM^ 500 LIZ Size Standard, 100bp, 139bp, 150bp, 160bp, 200bp, 250bp, 300bp, 340bp, 350bp, 400bp, 450bp, 490bp and 500bp respectively; peak 14 in Fig. 4(a), (b) and (c): 109bp, 211bp and 271bp respectively.


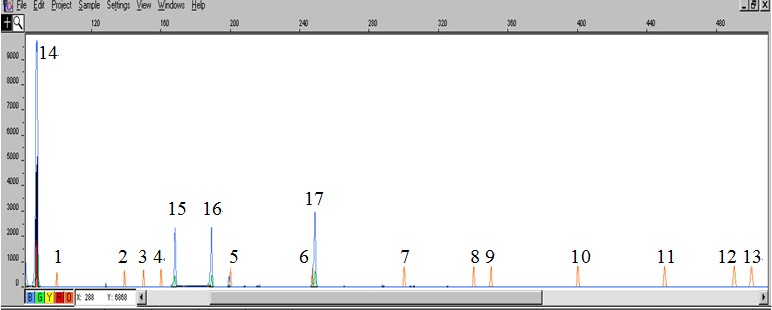


**Figure 11:** Results of 4-plex MLPA analysis with probe set for gene *Zein* and MON810, Nk603 and MON88017 events. The product was 91bp, 170bp, 191bp and 252bp long respectively. Peak 1-13: GeneScan^TM^ 500 LIZ Size Standard, 100bp, 139bp, 150bp, 160bp, 200bp, 250bp, 300bp, 340bp, 350bp, 400bp, 450bp, 490bp and 500bp respectively


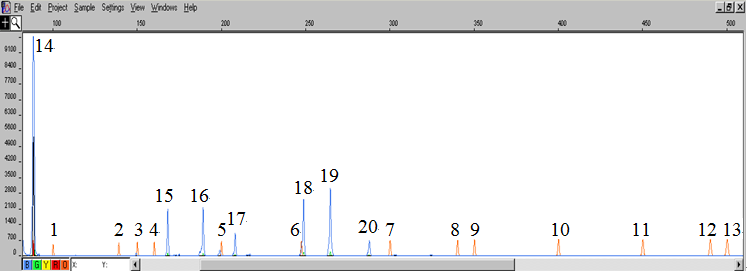


**Figure 12:** Results of 7-plex MLPA analysis with probe set for gene *Zein*, MON810, NK603, MON863, MON88017, MIR604 and GA21events. The product was 91bp, 170bp, 191bp, 211bp, 252bp, 271bp and 291bp long respectively. Peak 1-13: GeneScan^TM^ 500 LIZ Size Standard, 100bp, 139bp, 150bp, 160bp, 200bp, 250bp, 300bp, 340bp, 350bp, 400bp, 450bp, 490bp and 500bp respectively


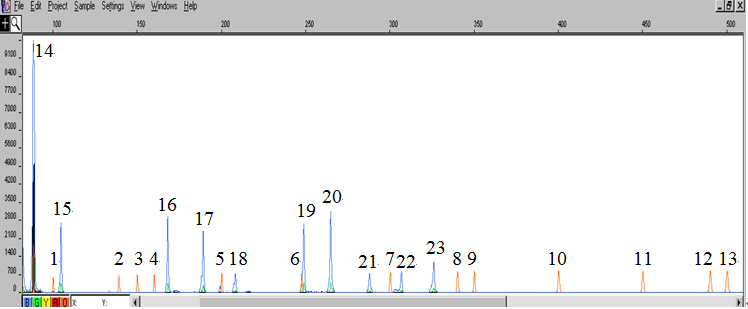


**Figure 13:** Results of 10-plex MLPA analysis with probe set for gene *Zein*, TC1507, MON810, NK603, MON863, MON88017, MIR604, GA21, BT11 and 59122 events. The product was 91bp, 109bp, 170bp, 191bp, 211bp, 252bp, 271bp, 291bp, 310bp and 330bp long respectively. Peak 1-13: GeneScan^TM^ 500 LIZ Size Standard, 100bp, 139bp, 150bp, 160bp, 200bp, 250bp, 300bp, 340bp, 350bp, 400bp, 450bp, 490bp and 500bp respectively
